# Pantothenate kinase-associated neurodegeneration

**DOI:** 10.17712/nsj.2017.2.20170197

**Published:** 2017-04

**Authors:** Khalid Hundallah, Afnan Al Hakeem

**Affiliations:** *From the Division of Neurology, Department of Pediatrics, Prince Sultan Military Medical City, Riyadh, Kingdom of Saudi Arabia*

## Case Presentation

9-years-old girl, previously healthy, presented with progressive dystonia. Clinically, she have dysarthria and spastic gait. Her parents are first-degree cousins. She have a paternal cousins who have a similar symptoms started 3 years ago, currently he is bed ridden.


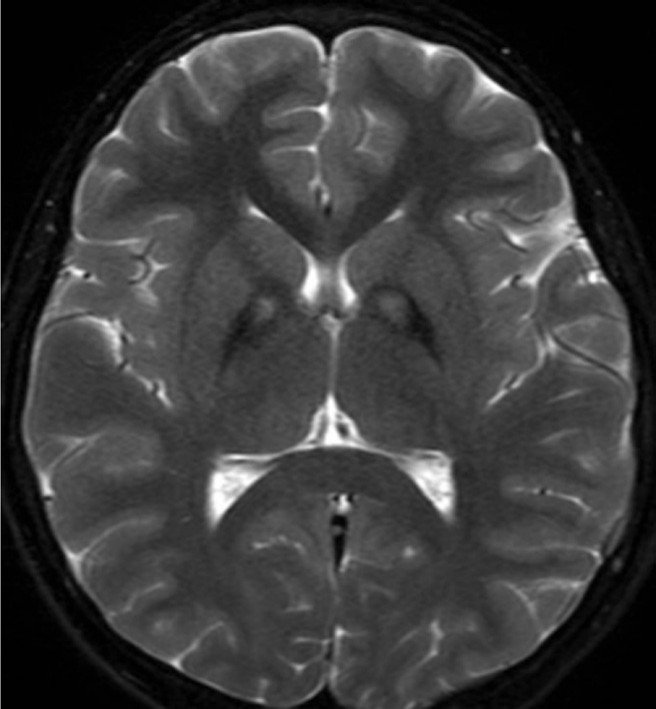


**Questions**

1. Her MRI brain is consistent with which of the following?
  - Pelizaeus merzbacher disease
  - Wilson disease
  - Pantothenate kinase-associated neurodegeneration (PKAN)
  - Sjogern larsson syndrome
2. Pantothenate kinase-associated neurodegeneration is caused by mutation in which of the following genes?
  - PANK2
  - ATP7B
  - PLP1
  - ADAT3
3. What is the underlying pathology in PKAN?
  - Copper deposition
  - Iron deposition
  - Post-infectious squeal
  - Calcium deposition
4. Which of the following can be used to manage dystonia in patients with PKAN?
  - Intramuscular botulinum toxin
  - Oral baclofen and trihexyphenidyl
  - Ablative pallidotomy or thalmotomy and deep brain stimulation.
  - All the above
5. In treating dystonia, what is the target brain structure in deep brain stimulation?
  - Ventrolateral thalamus
  - Putamen
  - Globus pallidus interna
  - Cerebellum

## Answers & Discussion

1. **c**
  MRI brain shows bilaterally symmetrical, hyperintense signal changes in the anterior medial globus pallidus, with surrounding hypointensity in the globus pallidus, on T2-weighted images. These imaging features have been termed the “eye-of-the-tiger sign.” The hyperintensity represents pathologic changes, including gliosis, demyelination, neuronal loss, and axonal swelling. The surrounding hypointensity is due to loss of signal secondary to iron deposition. eye-of-the-tiger sign is key diagnostic feature of PKAN (classical but not 100% pathognomonic).^1^
2. **a**
  Pantothenate kinase-associated neurodegeneration, formerly called Hallervorden-Spatz Disease, is an autosomal recessive disorder, characterized by progressive extrapyramidal dysfunction and dementia. A mutation in the pantothenate kinase (PANK2) gene on band 20p13 has been described in patients with PKAN. A mutant PANK2 gene located at the chromosomal locus 20p13-p12.3 causes the disorder.^2^
3. **b**
  PKAN is a subtype of Neurodegeneration with Brain Iron Accumulation (NBIA). NBIA comprises a group of inherited neurodegenerative disorders that are characterized symptomatically by extrapyramidal movement disorders and pathologically by abnormal iron accumulation in deep basal ganglia.^3^
4. **a**
  Pharmacologic and surgical interventions have focused on palliation of symptoms. Symptomatic treatment is aimed primarily at the dystonia, which can be profoundly debilitating and distressing to the affected individual and caregivers. Some patients benefit from benzodiazepines, anticholinergic medications, botulinum-toxin, baclofen, or intrathecal baclofen. Stereotactic pallidotomy have been tried in patients with severe dystonia, resulting in partial relief of symptoms. Deep brain stimulation is being used clinically with increasing frequency with promising results.^4^
5. **c**
  Deep brain stimulation has become a common treatment for primary dystonia, it is also being used more frequently to attempt to treat secondary dystonia seen in PKAN. It involves implantation of a neurostimulator which sends electrical impulses, through implanted electrodes to globus pallidus internus.^5^
